# Sexual education in the school setting: an overview of the Italian situation

**DOI:** 10.1093/eurpub/ckac131.441

**Published:** 2022-10-25

**Authors:** T Cappelletti, G Lo Moro, HSMA Elhadidy, F Bert, G Scaioli, R Siliquini

**Affiliations:** 1 Department of Public Health Sciences, University of Turin, Turin, Italy; 2 A.O.U. City of Health and Science of Turin, University of Turin, Turin, Italy

## Abstract

**Background:**

There is an important interaction between sexuality and many life factors. A good sexual education at school can improve knowledge and behaviour in young people giving them a healthy sexual life. Unlike most European countries, sexual education in Italy is not compulsory in the school curriculum, so this study explored the Italian regional initiatives realised over a span of 15 years.

**Methods:**

A review of grey literature was conducted on Public Health Administrations/Regions websites of each Italian region, focusing on official documents containing training catalogues for schools. The search was conducted in December 2021, including documents produced between 2006 and 2021. We used the topics recommended by UNESCO as quality markers for the projects found.

**Results:**

Among the 20 Italian regions, 12 had at least one programme. A total of 39 projects were found. All UNESCO topics were covered, with notable differences between north, centre and south of Italy, which had the lowest number of projects. Most of the projects (23) were carried out only once, the others were repeated at least for two years in a row. Contraception, along with love, marriage, partnerships, and family, were the main topics discussed during sexual education programs in schools (92%), followed by biological aspects, body awareness, and anatomy (83%); birth, disability, human rights, and online media were less common (33%). Regarding the target, disability, human rights, and mutual consent were deepened only for middle and high school students.

**Conclusions:**

Considering the fundamental importance of sexual education, there is an important lack in promotion and planning in Italy. There is a large discrepancy between the northern and southern regions and it is necessary to implement and standardize the offer of sexual education programs in schools.

**Key messages:**

• Unlike the European average, In Italy there is an important lack on sexual education, whit large discrepancies between the northern and southern regions, putting the latter at disadvantage.

• Contraception, marriage, couples, and family were the main topics discussed during sexual education programs in schools; birth, disability, human rights, and online media the least.

